# Molecular Epidemiology of Measles Viruses in the United States, 1997–2001

**DOI:** 10.3201/eid0809.020206

**Published:** 2002-09

**Authors:** Paul A. Rota, Stephanie L. Liffick, Jennifer S. Rota, Russell S. Katz, Susan Redd, Mark Papania, William J. Bellini

**Affiliations:** * Centers for Disease Control and Prevention, Atlanta, Georgia, USA

**Keywords:** virologic surveillance, molecular epidemiology, measles

## Abstract

From 1997 to 2001, sequence data from 55 clinical specimens were obtained from confirmed measles cases in the United States, representing 21 outbreaks and 34 sporadic cases. Sequence analysis indicated the presence of 11 of the recognized genotypes. The most common genotypes detected were genotype D6, usually identified from imported cases from Europe, and genotype D5, associated with importations from Japan. A number of viruses belonging to genotype D4 were imported from India and Pakistan. Overall, viral genotypes were determined for 13 chains of transmission with an unknown source of virus, and seven different genotypes were identified. Therefore, the diversity of Measles virus genotypes observed in the United States from 1997 to 2001 reflected multiple imported sources of virus and indicated that no strain of measles is endemic in the United States.

 An important component of laboratory surveillance for measles is the genetic characterization of wild-type viruses (1). This genetic information provides a powerful adjunct to standard epidemiologic data for describing the transmission pathways of Measles virus (MeV). Molecular epidemiology supports classical epidemiology in cases for which the source of imported MeV is known by confirming that the viral genotype obtained is consistent with the genotype known to be circulating in the country or region from which the case was imported. Molecular epidemiology fills in the gaps of information when classical epidemiology fails to discover the source of MeV, by providing a likely source on the basis of the genotypic information.

Monitoring the pattern of measles genotypes in an area can help document the effectiveness of control measures. For example, in areas that have endemic transmission of measles, virologic surveillance of cases detects a limited number of genotypes. On the other hand, in areas where endemic transmission of virus has been interrupted, a variety of genotypes are detected, reflecting the multiple sources of imported viruses. Virologic surveillance has already been used to help document the interruption of transmission of measles in the United States (2–4) and Australia (5). In addition, genetic analysis of viruses provides a means to differentiate vaccine-associated cases of measles from cases caused by infection with wild-type virus. Current surveillance protocols call for the collection of appropriate specimens for virologic surveillance during all phases of measles control. For countries such as the United States in the elimination phase of measles control, the goal is to collect a specimen for viral isolation along with a serum sample at first contact with each suspected case.

 Genetic characterization of wild-type MeV is based on sequence analysis of two variable regions on the viral genome. The targets for molecular epidemiologic studies are the 450 nucleotides coding for the 150 amino acids comprising the COOH-terminus of the nucleoprotein and the entire protein-coding region of the hemagglutinin gene. Based on these sequences, a number of genotypes have been identified (2,3,5–18). The World Health Organization (WHO) recognizes 20 genotypes and one proposed genotype (19–21), including several new genotypes that have been identified in the last 3 years (14,15,21–23). The prototype (Edmonston) strain of measles as well as all the currently used measles vaccines are in genotype A (21).

 The purpose of this report is to describe the genetic characteristics of wild-type measles viruses isolated in the United States during 1997–2001. Overall, the results show a pattern consistent with the continued interruption of endemic transmission. Viruses representing several of the recently described genotypes were associated with imported cases in the United States, and this information has increased our understanding of the degree of genetic diversity in wild-type measles viruses.

## Methods and Materials

Throat swabs and urine sediments were obtained from patients with serologically confirmed cases of measles. Clinical specimens were inoculated onto B95a cells (24), and the cells were observed for cytopathic effect (CPE). Inoculated cells were blind-passaged up to three times before being discarded. Cells were harvested when the CPE was maximal. Total cellular RNA was extracted from infected B95a cells or directly from clinical specimens, if virus isolation was not successful, by the guanidinium acid-phenol method (25). cDNA corresponding to the 565 nucleotides coding for the COOH-terminus of the nucleoprotein (N) and the full-length open reading frame for the hemagglutinin (H) gene were synthesized by using Avian myeloblastosis virus reverse transcriptase and amplified by polymerase chain reaction (PCR), as described previously (2,3). Sequences of the PCR products were derived by automated sequencing with the BigDye terminator chemistry according to the manufacturer’s protocol (Perkin Elmer-Applied Biosystems, Foster City, CA), and reaction products were analyzed on an automatic sequencer (ABI 373, ABI 3100, Perkin Elmer-Applied Biosystems). Sequence data were analyzed by using version 10.1 of the Genetics Computer Group Sequence Analysis Software Package (Genetic Computer Group, Madison, WI) (26). Genotypes were assigned on the basis of phylogenetic analyses performed by using PAUP version 4.0 (27). All phenograms were drawn as unrooted trees. Sequences described were deposited in GenBank under accession numbers AY037009–AY037048.

MeV genotype information was combined with epidemiologic information for each confirmed measles case. The epidemiologic information was gathered through case investigation by state and local health departments and reported to the Centers for Disease Control and Prevention through the National Notifiable Diseases Surveillance System.

## Results

 For the years 1997–2001, the number of reported measles cases in the United States has been at record low levels. A total of 138 cases were reported in 1997, 100 cases/year in both 1998 and 1999, 86 cases in 2000 (4), and 108 cases for 2001 (provisional data). Most cases were imported from other countries or spread from imported cases.

During this 5-year period, 47 outbreaks consisting of >3 epidemiologically linked, confirmed cases occurred. These outbreaks were very small: only three outbreaks had >10 cases. Specimens for viral isolation were obtained from 31 (64%) of 48 outbreaks, and at least one virus was isolated in tissue culture from 19 of the 31 outbreaks from which specimens were submitted (Table 1). In addition, reverse transcriptase (RT)-PCR was successfully used to detect measles RNA in clinical specimens from two outbreaks when attempts to isolate virus failed (Table 1). Overall, genetic information was obtained from 21 (44%) measles outbreaks, while specimens from 10 outbreaks failed to yield a viral isolate or positive PCR signal. The inability to isolate MeV or detect measles RNA was due to failure to collect specimens within 5 days after the onset of rash or to improper storage. In addition to the outbreaks, 176 sporadic cases and 28 chains of transmission with 2 cases were reported during this period. Twenty-eight measles viruses were isolated from sporadic cases and two-case chains (Table 1); six additional specimens from sporadic cases were positive for measles RNA by RT-PCR.

**Table 1:** Summary of virologic surveillance for measles, United States, 1997–2001

Virus/specimena	Abbreviation	Date	Activity^b^	Genotype^a^	Source^c^
MVi/Michigan.USA/3.97	MI3-97	1/97	Sporadic case	D5	Japan
MVi/Minnesota.USA/13.97	MN13-97	3/97	Sporadic case	H2	Vietnam
MVi/Pennsylvannia.USA/17.97	PA17-97	4/97	Outbreak, 4 cases	D4	Unknown
MVi/Florida.USA/15.97/2	FL15-97	4/97	Sporadic case	D5	Japan
MVi/Texas.USA/18.97	TX18-97	4/97	Outbreak, 3 cases	D6	Europe
MVi/Florida.USA/19.97	FL19-97	5/97	Sporadic case	D6	Italy
MVi/California.USA/22.97	CA22-97	5/97	Sporadic case	C2	Germany
MVi/Nevada.USA/20.97	NV20-97	5/97	Sporadic case	H1	China
MVi/Masschusetts.USA/27.97	MA27-97	7/97	Outbreak, 4 cases	D6	Greece/Italy
MVi/Minnesota.USA/33.97	MN33-97	8/97	Outbreak, 5 cases	D6	Brazil
MVi/Massachusetts.MA.USA/30.97	MA30-97	7/97	Sporadic case	D6	Ukraine
MVi/Pennsylvannia.USA/28.97	PA28-97	7/97	Sporadic case	D6	Brazil
MVi/Washington.USA/31.97	WA31-97	7/97	Sporadic case	D6	Unknown
MVi/Massachusetts.USA/2.98	MA2-98	1/98	Sporadic case (1)	H1	China
MVi/Washington.USA/17.98	WA17-98	4/98	Sporadic case (1)	D6	Croatia
MVi/Indiana.USA/16.98	IN16-98	4/98	Outbreak: 3 cases	C2	Zimbabwe
MVi/NewYork.USA/16.98	NY16-98	4/98	Sporadic case (1)	D6	Germany
MVi/California.USA/23.98	CA23-98	6/98	Sporadic case (1)	D5	Japan
MVi/Vermont.USA/28.98	VT28-98	7/98	Sporadic case (1)	D6	Cyprus
MVi/Alaska.USA/32.98	AK32-98	9/98	Outbreak: 33 cases	D5	Japan
MVi/California.USA/7.99	CA7-99	2/99	Outbreak: 4 cases	D4	India
MVi/Washington.USA/12.99	WA12-99	3/99	Outbreak: 3 cases	D8	Italy
MVi/Conneticut.USA/16.99	CT16-99	4/99	Sporadic case (1)	D4	India
MVi/Texas.USA/28.99	TX28-99	7/99	Outbreak: 3 cases	D8	UK
MVi/Virginia.USA/37.99	VA37.99	9/99	Outbreak: 15 cases	D4	Kenya
MVi/Illinois.USA/50.99	IL50-99	12/99	Sporadic case	D7	Sweden
MVi/Michigan.USA/52.99	MI52-99	12/99	Outbreak: 6 cases	D6	UK
MVi/California.USA/1.00	CA1-00	1/00	Sporadic case	D4	Japan
MVi/NewYork.USA/7.00	NY7-00	2/00	Outbreak: 9 cases	D6	UK
MVi/Washington.USA/6.00	WA6-00	2/00	Sporadic case (1)	D5	Japan
MVi/California.USA/8.00	CA8-00	2/00	Sporadic case (1)	D6	Turkey
MVi/NewYork.USA/11.00	NY11-00	3/00	Sporadic case	D2	Ireland
MVi/Alaska.USA/16.00	AK16-00	4/00	Sporadic case	H2	Unknown
MVs/Hawaii.USA/20.00	HI20-00	5/00	Sporadic case	D5	Japan
MVs/California.USA/24.00	CA24-00	5/00	Outbreak (5)	G2	Unknown
MVs/Florida.USA/25.00	FL25-00	6/00	Sporadic case	H1	Unknown
MVi/Vermont.USA/24.00	VT24-00	6/00	Outbreak: 6 cases	D4	Ethiopia
MVi/Michigan.USA/35.00	MI35-00	9/00	Sporadic case	D5	Unknown
MVi/Kansas.USA/43.00	KS43-00	11/00	Sporadic case	D5	Unknown
MVi/California.USA/49.00	CA49-00	12/00	Outbreak: 3 cases	D3	Philippines
MVs/Washington.USA/2.01	WA2-01	1/01	Outbreak: 11 cases	H1	Korea
MVi/Maryland.USA/5.01	MD5-01	1/01	Outbreak: 4 cases	D3	Philippines
MVi/Massachusetts.USA/6.01	MA6-01	2/01	Outbreak: 3 cases	D4	Pakistan
MVs/Illinois.USA/5.01	IL5-01	2/01	Sporadic case	H1	Korea
MVi/Minnesota.USA/9.01	MN9-01	2/01	Sporadic case	H1	Unknown
MVi/Washington.USA/9.01	WA9-01	2/01	Sporadic case	H1	China
MVi/California.USA/13.01	CA13-01	3/01	Sporadic (1)	D5	Unknown
MVs/Hawaii.USA/22.01	HI22-01	5/01	Sporadic case	D5	Unknown
MVs/California.USA/31.01	CA31-01	7/01	Sporadic case	D5	Japan
MVi/NewYork.USA/28.01	NY28-01	7/01	Outbreak: 4 cases	D5	Japan
MVi/Minnesota.USA/35.01	MN35-01	8/01	Sporadic case	D4	Kenya
MVi/Minnesota.USA/36.01	MN36-01	9/01	Sporadic case	H2	Unknown
MVs/Arizona.USA/35.01	AZ35-01	9/01	Sporadic case	D7	Unknown
MVi/California.USA/38.01/1	CA38-01/1	9/01	Outbreak: 3 cases	D7	Europe
MVi/California.USA/38.01/2	CA38-01/2	9/01	Outbreak: 6 cases	D7	Unknown

^a^Strain name and genotypes as recognized by the World Health Organization (20). MVi indicates that sequence was obtained from a viral isolate; MVs indicate sequence was obtained directly from the specimen. ^b^For sporadic cases, the number of spread cases is indicated in parentheses. ^c^Source identified by standard epidemiologic techniques.

 Viruses representing 11 of the 20 genotypes recognized by WHO in 2001 (20) were isolated in the United States in 1997–2001 (Tables 1 and 2). Among the 13 chains of transmission for which an imported source of MeV was not detected by classical epidemiology, seven different genotypes were identified (Table 2).

**Table 2:** Frequency of detection of measles genotypes, United States, 1997–2001

Genotype	No.^a^	Source^b^
D6	13	European countries, Brazil, unknown
D5	12	Japan, unknown
D4	8	India, Kenya, Ethiopia, Pakistan, Japan, unknown
H1	7	China, Korea, unknown
C2	2	Germany, Zimbabwe
D8	2	Italy, United Kingdom
H2	3	Vietnam, unknown
D3	2	Philippines
D2	1	Ireland
D7	4	Sweden, Europe, unknown
G2	1	Unknown

^a^Number indicates the number of times a genotype was associated with either an outbreak or a case. ^b^Source of virus, if known, based on standard epidemiologic investigations.

Thirteen (24%) of the measles sequences detected from chains of transmission in the United States in 1997–2001 belonged to genotype D6, which has previously been associated with importation from European countries (2,3). Within the last 6 years, viruses in genotype D6 have been isolated in Brazil, Uruguay, Argentina, United Kingdom, Spain, Germany, Russia, Poland, and Luxembourg (9,10,16,28). During 1997–2001, genotype D6 viruses were imported into the United States from Italy, Greece, Ukraine, Croatia, Cyprus, and the United Kingdom. During 1997, viruses from genotype D6 were imported into the United States from the large measles outbreak that occurred in São Paulo, Brazil, and spread to other South American countries (29–33). The viruses that were isolated from cases imported from Brazil to Minnesota (MN33-97) and Pennsylvania (PA28-97) during 1997 had sequences identical to those obtained from measles viruses isolated during the outbreak in Brazil. Measles virus WA31-97 was isolated at the same time as MN33-97 and PA28-97 from a case in Washington with an unknown source of infection. The sequence of WA31-97 was identical to those of the two viruses imported from Brazil, suggesting that this virus may also have been imported from Brazil (Figure).

**Figure:**
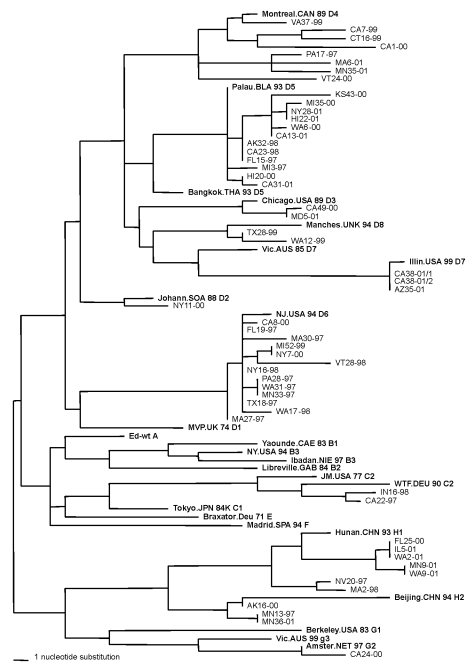
Genetic relationship between measles viruses isolated in the United States in 1997–2001 and the reference strains established by the World Health Organization (WHO) (20). Phylogenetic tree was based on the nucleotide sequences coding for the COOH-terminus of the nucleoprotein. Strain abbreviations are given in Table 1. Reference strains as established by WHO are shown in bold and designated by their genotype name. The length of the horizontal scale bar represents one nucleotide change.

 Twelve (22%) of the 55 sequences obtained from viral isolates or directly from clinical specimens were placed in genotype D5 (Tables 1 and 2). Genotype D5, along with D3, is one of the genotypes known to have endemic circulation in Japan (11). Epidemiologic investigations indicated that Japan was the source of infection for 6 of the 10 sporadic cases and two of the outbreaks. The largest outbreak in the United States during 1997–2001 occurred in Anchorage, Alaska, during 1998. The outbreak started 4 weeks after a case was imported from Japan to Anchorage. No direct epidemiologic link between this imported case and the outbreak was discovered. However, since the imported case and the outbreak occurred in the same place and within two generations of transmission, they were likely epidemiologically related. Although no viral specimens were received for the imported case, the sequence of the virus isolated during the outbreak was closely related to the sequences of viruses known to be circulating in Japan. Genotype D5 was also detected in specimens from a small outbreak that occurred in New York in July 2001 among a group of Japanese students who were visiting a university in New York City.

Viruses from genotype D4 were isolated from eight chains of transmission during 1997–2001; in seven of these chains, a foreign source of infection was identified (Tables 1 and 2). Importations from Kenya were associated with a 15-case outbreak in Virginia in 1999 and a sporadic case in Minnesota in 2001. At this time, no information is available about the genotypes of wild-type measles viruses circulating in Kenya, but a genotype D4 virus was imported into the United States from Kenya in 1996 (1). A genotype D4 virus imported from Ethiopia was responsible for a six-case outbreak in Vermont during 2000. In 2001, a genotype D4 virus was isolated from a small outbreak in Massachusetts, which was traced to a student from Pakistan. During 1999, two genotype D4 viruses were isolated from unlinked, imported cases from India. Genotype D4 is known to be circulating in Pakistan, Southern Africa, India, and Ethiopia (17,21,34). A genotype D4 virus (CA1-00) was isolated from a single imported case in California that was traced to Japan. This finding was unusual because genotype D4 viruses have never been detected in Japan despite extensive virologic surveillance.

Previous studies had shown that wild-type measles viruses isolated in the People’s Republic of China were members of a distinct genotype designated H1 (14). More recent information indicated that measles viruses circulating in both China and Vietnam were members of clade H but were sufficiently divergent from each other to be considered two separate genotypes, H1 and H2, respectively (35). Analysis of viruses imported from both China and Vietnam showed that these new genotype designations will be useful in epidemiologic surveillance. In 1997 and 1998, two viruses from genotype H1 were isolated from cases imported from China, and one virus in genotype H2 was isolated from a case imported from Vietnam. Two other viruses from genotype H2, AK16-00 and MN36-01, were isolated from sporadic cases that occurred in Alaska during 2000 and Minnesota during 2001, respectively. The sources could not be identified by standard epidemiologic investigation.

During 2001, genotype H1 viruses were isolated from an outbreak in Washington (WA2-01) and four sporadic cases in Florida (FL26-00), Washington (WA9-01), Illinois (IL5-01), and Minnesota (MN9-01). Concurrently, the Republic of Korea was experiencing a measles epidemic that began in 2000. Korea was identified as the source of the Washington outbreak and the sporadic case in Illinois. The sequences from the Washington outbreak and the sporadic case in Illinois were identical to those of genotype H1 viruses isolated in Korea (36). The person diagnosed with measles in Florida in 2000 had traveled to Los Angeles and Las Vegas shortly before onset of illness and probably was infected by a genotype H1 virus (FL26-00) while in transit. The sequence of FL26-00 was identical to that of the two viruses imported from Korea, suggesting that the source of this case may have also been the Korean outbreak (Figure). The sequence of the virus (WA9-01) from the sporadic case in Washington, which was imported from China, was very closely related to the sequences of the Korean viruses. This finding suggests that the genotype H1 viruses circulating in both China and Korea are closely related (Figure). The sequence of the virus identified in the Minnesota case (MN9-01) with an unknown source was identical to that of the Washington case imported from China (WA9-01).

Viruses from genotype C2 were isolated from two imported cases in the United States. Detecting a genotype C2 virus (CA2-97) in association with an importation from Germany was not unusual since genotype C2 viruses have frequently been detected in Europe (21), but the isolation of a C2 virus (IN16-98) from a patient returning from Zimbabwe was unexpected. No viral isolates from Zimbabwe have been characterized, and genotype C2 viruses have not been detected in any countries in southern Africa. In imported cases, the source of infection is usually assumed to be the country in which the person was traveling during the incubation period. However, this patient may have been infected while in transit from Africa to the United States via Europe. In fact, in this case, rash onset was 14 days after completion of travel, suggesting that the infection occurred near the end of the trip.

 Measles viruses in genotype D2 are known to circulate in both South Africa and Zambia (13,17). A genotype D2 virus was obtained from a single case imported into the United States from Dublin, Ireland, in 2000 (NY11-00). Although genotype D2 viruses are probably not endemic in Ireland, genotype D2 viruses were isolated during an outbreak that occurred in an immigrant community in Dublin that had low vaccination coverage. The sequence of NY11-00 was identical to the sequence of a virus isolated during the Irish outbreak (37).

 Viruses from genotype D3, the genotype associated with the resurgence of measles in the United States in 1989–1991, were detected from small outbreaks in California and Maryland in December 2000 and January 2001, respectively. In both outbreaks, the source of infection was the Philippines. The only other genotype D3 virus detected in the United States after 1993 was also imported from the Philippines to California in 1996 (3).

 Six of the sequences obtained from cases in 1999 and 2001 (TX28-99, WA12-99, IL50-99, CA38-01/1, AZ35-01, CA38-01/2) were closely related to the recently recognized genotypes D7 and D8 (Figure). A retrospective study showed that viruses from genotype D7 were isolated in Australia as early as 1985 (5). The sequences of IL50-99, CA38-01/1, AZ35-01, and CA38-01/2 were most closely related to the sequences from the Australian genotype D7 viruses. Interestingly, a virus isolated in Canada in 2000 from a case with a travel history to Mexico had a sequence nearly identical to that of IL50-99 (a WHO reference strain for genotype D7), which was imported into the United States from Sweden (38). In 2001, genotype D7 viruses (CA38-01/1, CA38-01/2, AZ35-01) were associated with two small outbreaks in California and a sporadic case in Arizona. One of the outbreaks in California had a European source (Table 1). Genotype D7 viruses were isolated from cases that were imported into El Salvador from Europe (39) as well as from outbreaks in Germany (40), suggesting that D7 may be another endemic European genotype. The two viral isolates from genotype D8, TX28-99 and WA12-99, had identical H gene sequences, although the sources of importation were different. The sequences of these viruses were most closely related to that of a virus isolated in the United Kingdom in 1994 (10), which has been designated the reference strain for genotype D8. Viruses in genotype D8 have been detected in Ethiopia and Nepal in 1998 and 1999, respectively (34,41).

 The source of a small outbreak in Los Angeles, California, during May–June 2000 was never identified. While no viral isolate was obtained, measles RNA was detected in some of the clinical samples. Sequences of the PCR product showed that a virus in genotype G2 was responsible for the outbreak. Viruses in genotype G2 are known to be circulating in Indonesia and Malaysia and were associated with importation of virus from Indonesia to the Netherlands (22,23). However, virologic surveillance has not been established in most areas of Asia, and genotype G2 viruses may be circulating in countries other than Malaysia and Indonesia.

## Discussion

 This study demonstrates the utility of virologic surveillance, especially for countries in the elimination phase of measles control. Sequence data obtained from 55 viral isolates or clinical specimens from measles cases in the United States during 1997–2001 indicated that 11 genotypes of virus were represented. No genotype was detected in a consistent pattern that would indicate endemic transmission. Rather, the diversity of genotypes reflects multiple, imported sources of measles virus. When the source of virus was identified by standard epidemiologic investigation, virologic surveillance helped to confirm the source of the virus and to build a genetic database of viral sequences associated with imported cases from different areas. Virologic surveillance was especially useful for characterizing 13 chains of infection in which the source of infection could not be identified by standard epidemiologic methods. Seven different genotypes were detected in these 13 chains, indicating multiple sources of infection. This finding suggests that these unknown source cases were the result of imported virus and not caused by circulation of an endemic genotype. These results underscore the need to improve the mechanism for obtaining appropriate specimens for viral isolation from all suspected cases, especially outbreak-associated cases. In countries that are in the elimination phase of measles control, obtaining specimens for viral isolation at first contact with all suspected measles cases is important.

Viruses isolated during the resurgence of measles in the United States in 1989–1991 were all in genotype D3, suggesting that D3 viruses had spread throughout the entire country (2,3). Following the resurgence, both standard epidemiologic and virologic surveillance indicated that endemic transmission was interrupted in the United States in 1993 (2,3,42,43). After 1993, only three viruses from genotype D3 were isolated in the United States, and all three were the result of importations from the Philippines (3). The pattern of MeV genotypes observed in the United States in 1993–2001 suggests an absence of indigenous transmission of virus since no genotype was consistently isolated. The pattern of viral genotypes reported for the United States after 1993 has been observed in other areas of the world that have good virologic surveillance and have achieved a high level of measles control. In Canada, the pattern of viral genotypes detected over the last 10 years has been very similar to the pattern in the United States (38). Likewise, virologic surveillance in Victoria, Australia, over a 25-year period suggested that repeated importation of multiple genotypes had occurred (5), and a similar pattern has been reported for the United Kingdom (10). In contrast, in countries that still have indigenous transmission of measles, only a limited number of genotypes are circulating (14,15).

 The tremendous reduction of measles cases in the United States after 1991was due in part to the successful measles control and elimination program initiated by the Pan American Health Organization (PAHO) in the early 1990s. However, during 1997, Brazil had a resurgence of measles, with nearly 50,000 cases reported (44,45). The outbreaks in Brazil eventually spread to several other South American countries. Genetically homogeneous viruses in genotype D6 viruses were associated with all the recent measles activity in South America except for two cases imported into El Salvador in 2001. In the last 2 years, PAHO has reported a record low number of measles cases in the Americas and <2,000 cases were reported for the year 2000 (46); most cases occurred in the Dominican Republic and Haiti. Virologic surveillance will play a key role in documenting the elimination of endemic transmission of the genotype D6 viruses in South and Central America in the same manner that was used to document the elimination of the genotype D3 viruses in the United States. Efforts are under way to improve laboratory capacity and expand virologic surveillance in the Americas.

 Strengthening virologic surveillance activities will not only contribute to our understanding of the transmission pathways of MeV but also increase the sensitivity of measles diagnosis. As the prevalence of disease decreases, the positive predictive value of serologic testing also decreases. Having the laboratory capacity to detect MeV or viral RNA will be especially helpful for measles surveillance in areas where indigenous transmission has been interrupted and many of the suspected cases are sporadic.

The identification of new genotypes indicates that our current understanding of the extent of genetic diversity in measles strains throughout the world is incomplete. Virologic surveillance has not been initiated in many countries, and others are just beginning to collect appropriate samples. Virologic surveillance activities need to be initiated or expanded in countries that are in the outbreak control phase of measles control to obtain an accurate record of the pattern of endemic viral genotypes present in all areas of the world.
